# Use of Eflornithine (DFMO) in the Treatment of Early Alzheimer's Disease: A Compassionate Use, Single-Case Study

**DOI:** 10.3389/fnagi.2018.00060

**Published:** 2018-03-06

**Authors:** Jessica Alber, Kelly McGarry, Richard B. Noto, Peter J. Snyder

**Affiliations:** ^1^Department of Psychiatry and Human Behavior, Warren Alpert Medical School of Brown University & Rhode Island Hospital, Providence, RI, United States; ^2^Department of Neurology, Warren Alpert Medical School of Brown University, Providence, RI, United States; ^3^Division of General Internal Medicine, Department of Medicine, Warren Alpert Medical School of Brown University & Rhode Island Hospital, Providence, RI, United States; ^4^Department of Radiology, Warren Alpert Medical School of Brown University & Rhode Island Hospital, Providence, RI, United States; ^5^Interdisciplinary Neuroscience Program, University of Rhode Island, Kingston, RI, United States; ^6^Ryan Institute for Neurosciences, University of Rhode Island, Kingston, RI, United States

**Keywords:** mild cognitive impairment, Alzheimer's disease, case study, eflornithine (DFMO), amyloid pathology

## Abstract

**Background:** Recent genome-wide association screening (GWAS) studies have linked Alzheimer's disease (AD) neuropathology to gene networks that regulate immune function. Kan et al. recently reported that *Arg1* (an anti-inflammatory gene that codes for arginase-1) is expressed in parts of the brain associated with amyloidosis prior to the onset of neuronal loss, suggesting that chronic brain arginine deprivation promotes AD-related neuropathology. They blocked arginine catabolism in their mouse AD model by administration of eflornithine (DFMO) to juvenile animals, effectively blocking the expression of AD-related amyloid pathology as the mice aged. We report results from a single-case study in which DFMO was administered, for the first time, in an attempt to slow progression of AD in a single woman with multi-domain, amnestic MCI who was unable to tolerate an acetylcholinesterase inhibitor.

**Methods:** Patient C.S. is a 74-year old female with a 5-year history of cognitive decline who was placed on DFMO (500 mg b.i.d.) for 12 months, with amyloid PET scans (baseline and 12-months), APOE genotyping and neuropsychological exams at baseline, 3, 9, and 12 months.

**Results:** C.S. suffered continued cognitive decline over 12 months, including progressive worsening of orientation, social functions and ability to engage in IADL's. She also showed progressive decline on measures of episodic memory and executive function. Florbetapir PET imaging yielded elevated total neocortical SUVr scores at both baseline (SUVr = 1.55) and at 12 months (SUVr = 1.69).

**Conclusions:** We report a first attempt at using DFMO to slow AD progression. This 12-month single-case trial did not halt continued amyloidosis nor cognitive decline. Although this trial was predicated on data reported by Kan et al. (2015) showing that DFMO administered to *juvenile* AD-prone mice led to diminished amyloid aggregation, this attempt to treat an older mild AD patient may not be a fair test of Kan et al.'s model and results. A future trial might seek to block amyloidosis in young adults who are autosomal gene carriers for early onset AD, or perhaps in adults who are very clearly in the pre-clinical disease stage.

**Trial Registration:** This trial was registered as a Compassionate Use IND #128888 with the United States Food and Drug Administration (FDA).

## Background

Alzheimer's disease (AD) is a neurodegenerative condition associated with the accumulation of parenchymal β-amyloid (Aβ) and hyperphosphorylated tau tangles in the neurons. The pathogenesis of AD is a complicated process that occurs as a result of a combination of downstream interactions between DNA-sequence variants and non-genetic (biological risk factors such as vascular health and psychosocial risks) contributors that act through molecular networks to affect disease risk (Schadt, 2009). The exact mechanism of AD pathogenesis remains unknown despite decades of careful research. To date, there are no preventative or disease-modifying AD therapies, despite the projected more than doubling of the incidence of AD by the year 2050 (Alzheimer's Association Strategic Plan, 2017). Currently, one dominant etiologic model of AD is the referred to as the “amyloid hypothesis” (Hardy and Allsop, 1991; Selkoe, 1991; Hardy and Higgins, 1992; Jack et al., 2013), which states that parenchymal Aβ builds up decades prior to the onset of clinical symptoms, resulting in a silent period termed preclinical AD (Sperling et al., 2011), followed by neurodegeneration and the accumulation of hyperphosphorylated tau. Therapeutic interventions now focus on this preclinical AD period, with the aim of eliminating the accumulation of parenchymal Aβ early in the pathological process of the disease.

The apolipoprotein E (APOE) gene is the AD susceptibility gene that has been most extensively researched, and accounts for an estimated 30% of the genetic variability in AD (Corder et al., 1993; Saunders et al., 1993). Despite recent developments indicating other genetic contributions to AD such as APP and TREM2 (Jonsson et al., 2012, 2013; Guerreiro et al., 2013), a large proportion of the genetic variance in AD remains unaccounted for. In addition to APOE, genome wide association studies (GWAS) have linked AD pathology to gene networks that regulate immune function and microglial expression (Kamboh et al., 2012; Zhang et al., 2013).

Most animal models of AD involve transgenic expression of mutated amyloid precursor protein (APP), which leads to parenchymal Aβ deposition but does not usually invoke tau pathology and associated neuronal cell death. Consequently, these models have been criticized as incomplete models of AD pathology (Irizarry et al., 1997; Radde et al., 2008). In a recently developed CVN-AD mouse model, immune-mediated nitric oxide (iNOS) was lowered to mimic human levels, resulting in a model that demonstrates the complete pathological course of AD, including parenchymal amyloidosis, gradual spread of hyperphosphorlyated tau, episodic memory impairment, and significant hippocampal neuronal degeneration (Colton et al., 2008, 2014; Wilcock et al., 2014). These mice are mNos2 -deficient and transgenic for the Swedish K670N/M671L vasculotropic Dutch/Iowa E693Q/D694N mutant (APPSwDI) APP (Kan et al., 2015), which prevents the expression of inducible nitric oxide synthase protein, thus lowering the level of nitric oxide production (for a detailed description of this model, see Colton et al., 2008, 2014; Wilcock et al., 2014).

Using this model, Kan et al first found that these CVN-AD mice followed the pathological progression of human AD (Kan et al., 2015). In an additional study, they found that *Arg1* (an anti-inflammatory gene that codes for arginase1, an arginine utilization enzyme) was expressed in the parts of the animal brain with high levels of amyloidosis prior to the onset of neuronal degeneration and cell death, an indication that chronic brain arginine deprivation could be the cause of neuronal cell death in AD, and the subsequent cascade of AD pathology. Based on this information, they administered eflornithine (DFMO; an arginase inhibitor) to juvenile animals, which effectively blocked the expression of Aβ and other AD related pathology, immunosuppression, and spatial memory impairments. Moreover, the DFMO mice had higher arginine expression in the hippocampus and subiculum, areas that are associated with early neuronal loss in AD pathology. In other words, inhibition of abnormal arginine utilization and resultant arginine expression resulted in marked improvement in pathology and cognitive function when administered to juvenile CVN-AD mice (Kan et al., 2015).

In response to a special request by a family member of a patient, who happened to be a pediatric oncologist with clinical experience in the use of DFMO for the treatment of pediatric neuroblastomas and who also was aware of the Kan et al. (2015) publication, one of us (K.M.) applied for—and received approval for—a compassionate use Investigation New Drug (IND) application to administer DFMO to a single woman with multi-domain, amnestic mild cognitive impairment (MCI) who was unable to tolerate an acetylcholinesterase inhibitor. DFMO is an Food and Drug Administration (FDA) approved treatment of Trypanosoma brucei gambiense encephalitis (“African sleeping sickness”), has been used in clinical trials for neuroblastoma in children, and has a favorable safety and tolerability profile. It is unknown whether or not DFMO would slow or reverse amyloid pathology, or improve cognitive outcomes in a human subject with MCI.

## Methods

### Case presentation

The participant (C.S.) is a 74 year old, left-handed female with 16 years of education. Her past medical history is notable for hypertension, gastric reflux, osteoarthritis, headaches, benign essential tremor, glaucoma, anxiety, and depression. Concurrent medications upon initiation of the trial were omeprazole, sertraline, trazadone, Miralax, calcium, fish oil, and baby aspirin. The researchers had access to the participants' medical records and previous neuropsychological evaluations in 2011 and 2013, which were negative for neurodegenerative disease diagnosis but noted mild cognitive changes due to anxiety and depression. Family history was positive for Alzheimer's disease in both parents, with onset in her father in the early 80s and onset in her mother in mid-80's.

The participant presented with a 5-year gradually progressive history of cognitive decline and in 2015 she received a diagnosis of multi-domain amnestic MCI due to possible Alzheimer's disease. Symptoms included disorientation to time, repetitive speech, mild word finding difficulty, difficulty following conversations, confusability, and difficulties with attention and concentration. The patient had mild, stable symptoms of depression and anxiety and had very minor dilapidation of instrumental activities of daily living (IADL's) such as driving and financial management. The patient had also become more socially withdrawn as a result of conversational difficulties and lost ~25 pounds over the 2 years preceding presentation. After diagnosis, the participant began a regimen of donepezil 5 mg, but this was discontinued due to GI problems and excessive weight loss. The patient's inability to tolerate a cholinesterase inhibitor resulted in application for a compassionate use IND for eflornithine (DFMO).

### Procedure

#### Screening

Baseline screening assessments included medical history, physical exam, vital signs (blood pressure, pulse, temperature), and complete blood count (CBC) with differential and comprehensive metabolic panel (CMP). Prior to baseline dosing, the participant completed an amyloid PET scan and neuropsychological evaluation.

#### Treatment

The participant was administered eflornithine (DFMO) 500 mg (2 tabs) PO BID (morning and evening), concomitant with safe therapeutic dosing in the United States for colon cancer prevention and neuroblastoma. DFMO was prescribed by KM (primary care physician) as oral capsules, taken morning and evening with water. Dosing and medication instructions were explained verbally to the participant and her caregiver, and written instructions were also provided regarding dosing and timing of dose.

#### Safety monitoring

Safety monitoring involved a CBC with differential and a CMP every 2 weeks for four results, then once a month for four results, then once every 2 months for four results. Additionally, repeat medical history, physical exam, vital signs, and blood tests were conducted once per month for 6 months, and then once every 2 months for 6 months.

### Outcome measures

#### Neuropsychological evaluation

Comprehensive neuropsychological evaluation was conducted at baseline, 3, 9, and 12 months. Neuropsychological evaluation included the domains and tests described in Table 1. A clinical interview was conducted with the participant and her caregiver prior to each neuropsychological session to assess changes in medications, medical history, changes in health, functional changes, and current symptoms of depression/anxiety. Clinical interview and neuropsychological tests were administered by a post-doctoral research fellow with extensive training in neuropsychological assessment and reviewed by a licensed clinical neuropsychologist.

**Table 1:** Neuropsychological outcome measures used in a compassionate use IND trial of DFMO in a single case.

**Neuropsychological Domain**	**Tests Administered**
Global cognition	Mini Mental Status Exam (MMSE) (Folstein et al., 1975) Alzheimer's disease assessment scale-cognitive subscale (ADAS-Cog;11 item) (Rosen et al., 1993)
Verbal episodic memory	Logical Memory I (WMS-R) Immediate and Delayed Recall (Wechsler, 1997) International Shopping List Test (ISLT; Cogstate) (Lim et al., 2009)
Visual episodic memory	Groton Maze Learning Test (GMLT; Cogstate) delayed recall (Snyder et al., 2005; Fredrickson et al., 2008)
Language	Category Fluency (animals) (Borkowski et al., 1967)
Executive function	GMLT (Cogstate Ltd.) Learning (Snyder et al., 2005; Fredrickson et al., 2008) One Card-Learning Task (OCL; Cogstate Ltd.) (Maruff et al., 2013) Phonemic Fluency (FAS) (Borkowski et al., 1967; Tombaugh et al., 1999)
Working memory	Groton Maze Learning Test (GMLT; Cogstate Ltd.) (Snyder et al., 2005; Fredrickson et al., 2008) One-back Task (OBK; Cogstate Ltd.) (Maruff et al., 2013)
Processing speed	GMLT Chase Test (Cogstate Ltd.) (Snyder et al., 2005; Fredrickson et al., 2008)
Subjective cognitive complaints	Subjective Cognitive Decline (SCD) (Gifford et al., 2015)
Depression	Geriatric Depression Scale (GDS) (Yesavage and Sheikh, 1986)

Primary endpoints included reported decline in instrumental activities of daily living as assessed via clinical interview, change in score on the Mini Mental Status Examination (MMSE; Folstein et al., 1975), the ADAS-Cog (Rosen et al., 1993), delayed recall on Logical Memory (Wechsler, 1997), and change in amyloid PET burden as measured by Florbetapir PET scan. Secondary endpoints included change in score on the GMLT (Snyder et al., 2005; Fredrickson et al., 2008), verbal fluency (phonemic and category fluency; Borkowski et al., 1967; Tombaugh et al., 1999), OCL (Maruff et al., 2013), OBK (Maruff et al., 2013), and ISLT (Lim et al., 2009).

#### Amyloid PET imaging

Amyloid positron emission tomography (PET) imaging was conducted at baseline and after 12 months. At each scan, a 370 MBq (10 mCi ± 10%) bolus injection of ^18^F-florbetapir was administered intravenously. Approximately 50 min post-injection, a 20-min PET scan was performed with head computerized tomography (CT) scan for attenuation and correction purposes. Images were obtained using a 128 x 128 matrix reconstructed using iterative or row action maximization likelihood algorithms. PET standardized uptake value (SUV) data were summed and normalized to the whole cerebellum SUV, resulting in a region-to-cerbellum ratio termed SUV ratio (SUVr). SUVr calculation was performed using the MIMneuro software, with a normative database of 74 individuals (48 males, 26 females) between the ages of 18 and 50 years who met the eligibility criteria for inclusion; that is, individuals needed to be in good general health, score within normal range for standard clinical neurologic and cognitive testing, and have a negative amyloid scan on visual assessment (Clark et al., 2011). Amyloid positivity was confirmed by consensus of two board-certified radiologists who were also board certified in nuclear medicine.

## Results

Results from primary and secondary outcome measures are summarized in Table 2. For cognitive reasons, the participant was unable to complete the delayed recall portion of the GMLT in the baseline visit, exam 2 (3 months), and exam 3 (9 months). Additionally, she was unable to complete the GMLT reverse recall at the baseline visit and exam 2 (3 months).

**Table 2:** Scores on all outcome measures for patient CS, age 74, over 12 month DFMO treatment period.

**Measure**	**Exam 1 (baseline)**	**Exam 2 (3 months)**	**Exam 3 (9 Months)**	**Exam 4 (12 months)**
MMSE (/30)	26	26	24	22
ADAS-Cog (/70)	30	34	31	33
Logical Memory, Immediate Recall (/25)	9	4	6	6
Logical Memory, Delayed Recall (/25)	3	3	3	6
Phonemic Fluency (FAS)	38	41	22	32
Phonemic Fluency (animals)	9	9	5	9
GDS (/15)	4	1	2	2
Subjective Cognitive Decline (/9)	6	6.5	4	5
Chase Test mps	0.167	0.133	0.233	0.366
Chase Test total errors	14	11	5	8
Chase Test feedback errors	14	11	5	8
GMLT mps^a^	0.162	0.195	0.131	0.187
GMLT total errors^a^	31.67	37.75	30.00	37.00
GMLT feedback errors^a^	13.33	18.50	14.67	19.50
GMLT delayed recall: mps	^b^	^b^	^b^	0.125
GMLT delayed recall: total errors	^b^	^b^	^b^	30
GMLT delayed recall: feedback errors	^b^	^b^	^b^	20
GMLT reverse recall: mps	^b^	^b^	0.141	0.163
GMLT reverse recall: total errors	^b^	^b^	30	67
GMLT reverse recall: feedback errors	^b^	^b^	12	44
One Card Learning: accuracy	0.964	0.891	0.904	0.785
One Card Learning: total errors	26	32	31	41
One Back Task: accuracy		0.860	0.747	0.927
One Back Task: total Errors	6	20	21	18
ISLT Total Learning (3 trials, /36)	^c^	15	9	18
ISLT Delayed Recall (/12)	^c^	2	1	2
Florbetapir PET SUVr	1.55			1.69

a *Averaged across 5 learning trials*.

b *Patient did not complete for cognitive reasons*.

c *Patient did not complete for technological reasons*.

Throughout the 1 year trial period, the participant did not experience any clinical increase in depression or anxiety symptoms, assessed via self-report (GDS; Yesavage and Sheikh, 1986), caregiver report, and clinical interview. There were no significant changes in her level of subjective complaints, as measured by the Subjective Cognitive Decline scale (Gifford et al., 2015).

### Primary endpoints

**Figure 1:**
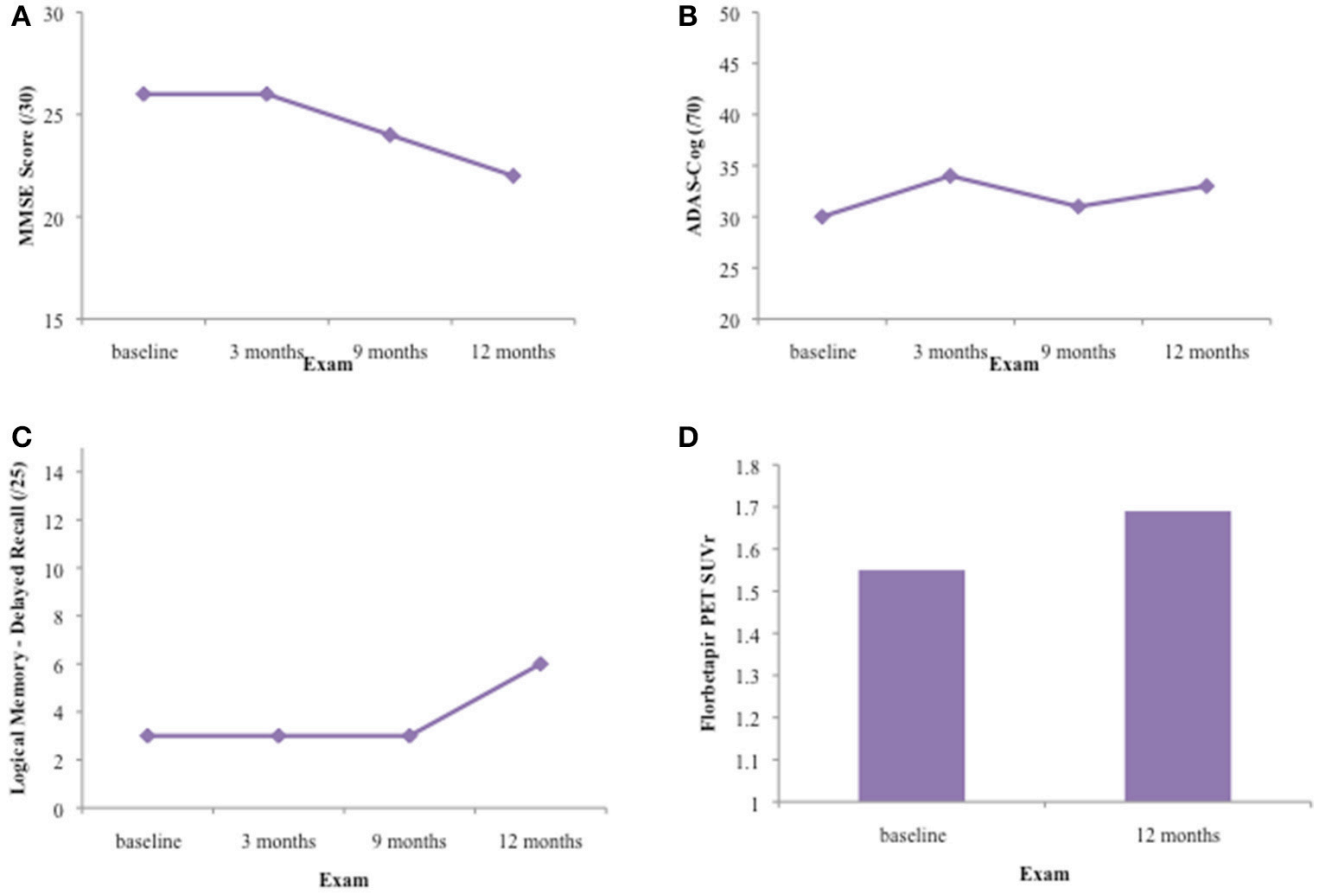
Results on primary outcome measures for patient CS over 12 months of DFMO therapy. **(A)** Results on the Mini-Mental Status Exam (MMSE)/30 at baseline, 3, 6, and 12 month exams. CS' MMSE score declined 4 points over the 12 month treatment period, from 26/30 to 22/30, indicating generalized cognitive decline. **(B)** Results on the Alzheimer's Disease Assessment Scale-Cognitive Subscale (ADAS-Cog) 11—item at baseline, 3, 6, and 12 month exams. Patient CS' ADAS-Cog score increased by 3 points over the 12 month treatment period, from 30/70 to 33/70. Higher scores on this test indicate increased cognitive impairment. **(C)** Results on the delayed recall portion of the Logical Memory stories at baseline, 3, 6, and 12 month exams. The patient showed impaired verbal learning and episodic memory throughout the 12 month treatment period. Although the patient recalled 6/25 items at the 12 month visit, this was not concurrent with results on other episodic memory tests such as the ADAS-Cog word list learning and recall (0/10 words at delayed recall) and the ISLT (2/12 shopping items at delayed recall). **(D)** SUVr on Florbetapir PET scan at baseline (1.55) and after 12 month treatment period (1.69).

The results for all four primary endpoints are summarized in Figure 1. There was no significant improvement in scores on the MMSE, ADAS-Cog, or Logical Memory delayed recall tests over the 1 year treatment period. The patient declined from an MMSE of 26/30 to an MMSE of 22/30 during this time. ADAS-Cog scores increased slightly during the treatment period, indicating cognitive decline. Learning and memory was severely impaired on the Logical Memory stories. Although the participant improved in terms of number of items recalled and percentage retained at the final visit, this was not corroborated by other memory tests, such as the word-list learning subtest of the ADAS-Cog and the ISLT.

The patient suffered significant functional decline during the 1 year treatment period. This included complete cessation of driving and inability to prepare complex meals or perform financial management activities. Additionally, the patient and/or her caregiver reported difficulty with orientation to time and place, multi-tasking, increased confusability, and inability to complete multi-step commands. The patient is no longer able to be home independently without supervision. Given this, the family has employed a caregiver to work with the patient once per week for respite care for her spouse, who is her full-time caretaker.

Her florbetapir PET scan was positive on visual read for both the baseline and 12 month visits. Her SUVr increased from 1.55 to 1.69 during the 1 year treatment period, indicating clinically increased neocortical amyloidosis commensurate with advancing Alzheimer's disease.

### Secondary endpoints

As seen in Table 2, there were no improvements on the patient's scores on any of the secondary endpoint measures.

### Efficacy assessment

Patient CS demonstrated continued symptomatic progression, both cognitively and functionally, over the 1 year treatment period on DFMO 500 mg p.o. BID, and there was an increase in cerebral amyloidosis as measured by Florbetapir PET imaging during this time. After the 1 year treatment period, she no longer met criteria for MCI, but rather met criteria for a diagnosis of mild-moderate dementia of the Alzheimer's type.

## Discussion

This report details what we believe is the first known administration of eflornithine (DFMO) as a therapeutic intended to slow mild AD progression in a single individual. The participant was a 74-year-old female with a diagnosis of multi-domain amnestic MCI, who could not tolerate cholinesterase inhibitors due to gastrointestinal irritation and subsequent weight loss. After 12 months of DFMO therapy, the participant declined on neuropsychological tests measuring global cognitive function, episodic memory, executive function, language, processing speed, and working memory. She showed no measurable improvement on the MMSE or ADAS-Cog. Additionally, the participants' cortical amyloidosis increased over the 12 month treatment period from a Florbetapir PET amyloid SUVr of 1.55 to 1.69. Functionally, she suffered decline in instrumental activities of daily living and some decline in basic activities of daily living. Taken together, these results indicate that treatment with DFMO was not effective in reducing or halting cortical amyloidosis, and did not lead to positive cognitive or functional outcomes in a patient with MCI. Over the 12 month period, the participant declined clinically and her diagnosis was changed from MCI to mild AD, commensurate with amyloid PET results, functional decline and neuropsychological test scores.

This 12 month, single-case study was predicated on a mouse model of AD (the CVN-AD model; Colton et al., 2008, 2014; Wilcock et al., 2014; Kan et al., 2015), which reduced the levels of iNOS in mice to mimic human levels, resulting in a mouse model that mirrored the course of human AD, beginning with asymptomatic amyloidosis and followed by hyperphosphorlyated tau, age-dependent hippocampal neuronal loss, and behavioral changes in episodic memory and executive function. This model showed that the pathological cascade of cortical amyloidosis in CVN-AD knockout mice is associated with increased arginine utilization, which is immune regulated, and decreased cerebral arginine bioavailability, particularly in areas affected early by AD such as the hippocampus. Additionally, they found that DFMO (a partial inhibitor of arginase, an enzyme that metabolizes arginine) administered to juvenile CVN-AD knockout mice led to reduced cortical amyloidosis and recovery of cognitive function (episodic memory) via the reduction of arginine catabolism by the enzyme arginase 1 (*Arg1*) and subsequent reduction of immunosuppression by cerebral microglia.

There are several potential factors contributing to the lack of efficacy of DFMO in this single case study. The primary contributor is that Kan et al. (Kan et al., 2015) administered DFMO beginning at 6–8 weeks of age in their CVN-AD knockout mice, and they had treatment dosage of 10 mg/kg via oral gavage 3 times per week for 14 weeks. CVN-AD knockout mice show Aβ accumulation at 6 weeks of age, hyperphosphorylated tau at 12 weeks of age, and behavior deficits at 24 weeks of age (Colton et al., 2014). The administration of DFMO in the CVN-AD knockout mice began concurrently with accumulation of cerebral Aβ, and the therapeutic window spanned the entire length of expected pathological changes in these juvenile mice. In our case, the participant had a Florbetapir PET SUVr of 1.55 at baseline, an indication of significant cortical amyloidosis. Visual inspection of the amyloid PET scan by a board-certified nuclear medicine specialist indicated a positive visual read, indicating increased uptake of Aβ in at least two separate neocortical areas. In our case, unlike Kan et al. (2015), the participant already had significant Aβ accumulation at baseline, which could have caused significant pathological changes resistant to change from DFMO therapy. We suspect that administration of DFMO in the very early stages of cerebral amyloidosis (preclinical AD) would have been a more accurate and reliable assessment of whether or not this therapeutic translates from mouse to human models.

There are still several unknowns regarding the mechanism by which immune suppression, particularly arginine deprivation, could prompt the initiation of the AD pathological cascade in this mouse model. We do know that there is direct evidence supporting that dysregulated arginine utilization may contribute to human AD pathology. Autopsy studies have shown an increase in arginase mRNA expression and enzymatic activity in human AD brain samples (Colton et al., 2006; Hansmannel et al., 2010; Liu et al., 2014). However, the exact cell type that is associated with increased arginine utilization and consumption remains unclear. There are several cell types capable of expressing arginase-1, and one particular cell type or a combination of these cell types could be driving arginine deprivation in AD. Kan et al. (2015) make a strong case for microglia, basing this assumption on the appearance of a particular type of microglial cell (CD11c+) very early in AD pathology that was highly correlated with AD pathology and concentrations of extracellular arginase. Complicating this story, arginine is the target of two opposing, immune regulated, enzyme systems: the iNOS pathway and the arginase pathway. These pathways co-exist, but competition between these two enzymes favors arginase in humans (Wu et al., 2009; Yi et al., 2009; Guo et al., 2012), and iNOS activity is decreased in human AD, resulting in upregulation of the arginase pathway (Colton et al., 2006; Yi et al., 2009; Liu et al., 2014). Further research elucidating the exact neurochemical mechanism by which immune arginine deprivation affects AD pathology will provide insight into next steps for potential translation to a usable therapeutic for preclinical AD patients.

More broadly, research has suggested a role for arginine in AD via interactions with atherosclerosis (Cooke and Creager, 1991; Boger et al., 1996), glucose metabolism (Schmidt et al., 1992; Smuckler et al., 2002), and neurogenesis (Ciani et al., 2006; Torroglosa et al., 2007). Further exploration of the role of arginine in these processes, which contribute to AD, will help to elucidate the peripheral effects of arginine on AD pathology.

## Concluding remarks

These unknowns notwithstanding, we have completed the first ever single case clinical trial of eflornithine (DFMO), a partial inhibitor of arginase, in a patient with multi-domain, amnestic MCI. After 12 months of DFMO treatment, the participant showed cognitive, functional, and pathological progression, and was diagnosed with mild AD. DFMO did not halt or reduce cerebral amyloidosis as measured by Florbetapir PET scan, and the participant's cognitive and functional outcomes decreased over a 12 month period at a rate commensurate with the normal course of neurodegenerative progression in AD. However, we believe that targeting immunosuppressive genes is still a reasonable scientific line of inquiry. Mouse models to date have targeted juvenile animals at the onset of cortical amyloidosis, while our participant already had a high cortical amyloid burden at onset of treatment as shown by Florbetapir PET scan.

Because DFMO is an FDA approved drug and has a favorable safety and tolerability profile, this therapeutic should be further explored as a potential target for preventative therapy in the very early stages of preclinical AD. Additionally, patients with autosomal dominant AD may be ideal targets for such therapy, as these patients are often relatively young, and age of onset tends to be in mid-late life and can be more accurately predicted.

## Ethics statement

This study was carried out in accordance with the recommendations of Institutional Review Board (IRB) at the Rhode Island Hospital (Providence, RI), and the patient described in this report provided written informed consent in accordance with the Declaration of Helsinki. This trial was registered as a Compassionate Use IND #128888 with the United States Food and Drug Administration (FDA).

## Author contributions

KM: Requested and acquired the compassionate use IND from FDA, administered medication, and completed all safety monitoring; JA and PS: Conducted all neuropsychological evaluations, analyzed all data, arranged amyloid PET scans and prepared the manuscript; RN: Analyzed amyloid PET results. All authors read and approved the final manuscript.

### Conflict of interest statement

The authors declare that the research was conducted in the absence of any commercial or financial relationships that could be construed as a potential conflict of interest.

## Abbreviations

Aβ: β-amyloid
AD: Alzheimer's disease
ADAS-Cog: Alzheimer's Disease Assessment Scale-Cognitive Subscale
ADL: activities of daily living
APOE: apolipoprotein
APP: amyloid precursor protein
Arg1: Arginase 1
BID: twice per day
CBC: complete blood count
CMP: comprehensive metabolic panel
CT: computerized tomography
DFMO: eflornithine
DNA: deoxyribonucleic acid
FDA: Food and Drug Administration
GDS: Geriatric Depression Scale
GMLT: Groton Maze Learning Test
GWAS: genome-wide association studies
IADL: instrumental activities of daily living
IND: Investigational New Drug
iNOS: immune-mediated nitric oxide
ISLT: International Shopping List Test
MCI: mild cognitive impairment
MMSE: Mini-Mental Status Exam
OBK: One-Back Task
OCL: One-Card Learning Task
PET: positron emission tomography
PO: by mouth
SCD: Subjective Cognitive Decline
SUV: standardized uptake value
SUVr: standard uptake value ratio
TREM-2: Triggering Receptor Expressed on Myeloid Cells-2.
